# Gingival Orofacial Granulomatosis Clinical and 2D/3D Microscopy Features after Orthodontic Therapy: A Pediatric Case Report

**DOI:** 10.3390/medicina59040673

**Published:** 2023-03-28

**Authors:** Chiara Cecchin-Albertoni, Laetitia Pieruccioni, Thibault Canceill, Robin Benetah, Jade Chaumont, Christophe Guissard, Paul Monsarrat, Philippe Kémoun, Mathieu Marty

**Affiliations:** 1 Oral Medicine Department and CHU de Toulouse, Competence Center of Oral Rare Diseases, Toulouse Institute of Oral Medicine and Science, CEDEX 9, 31062 Toulouse, Francemathieu.marty@univ-tlse3.fr (M.M.); 2 RESTORE Research Center, Université de Toulouse, INSERM, CNRS, EFS, ENVT, Batiment INCERE, 4bis Avenue Hubert Curien, 31100 Toulouse, France; 3 InCOMM (Intestine ClinicOmics Microbiota & Metabolism) UMR1297 Inserm, Université Toulouse III, French Institute of Metabolic and Cardiovascular Diseases (i2MC), CEDEX 4, 31432 Toulouse, France; 4 Artificial and Natural Intelligence Toulouse Institute ANITI, 31013 Toulouse, France; 5 LIRDEF, Faculty of Educational Sciences, Paul Valery University, CEDEX 5, 34199 Montpellier, France

**Keywords:** orofacial granulomatosis, orthodontic device, dental treatment, cheilitis, light sheet fluorescence microscopy

## Abstract

Orofacial granulomatosis (OFG) represents a heterogeneous group of rare orofacial diseases. When affecting gingiva, it appears as a chronic soft tissue inflammation, sometimes combined with the enlargement and swelling of other intraoral sites, including the lips. Gingival biopsy highlights noncaseating granulomatous inflammation, similar to that observed in Crohn’s disease and sarcoidosis. At present, the etiology of OFG remains uncertain, although the involvement of the genetic background and environmental triggers, such as oral conditions or therapies (including orthodontic treatment), has been suggested. The present study reports the results of a detailed clinical and 2D/3D microscopy investigation of a case of gingival orofacial granulomatosis in an 8-year-old male patient after orthodontic therapy. Intraoral examination showed an erythematous hyperplasia of the whole gingiva with a granular appearance occurring a few weeks after the installation of a quad-helix. Peri-oral inspection revealed upper labial swelling and angular cheilitis. General investigations did not report ongoing extra-oral disturbances with the exception of a weakly positive *anti-Saccharomyces cerevicae* IgG auto-antibody. Two- and three-dimensional microscopic investigations confirmed the presence of gingival orofacial granulomatosis. Daily corticoid mouthwashes over a period of 3 months resulted in a slight improvement in clinical signs, despite an intermittent inflammation recurrence. This study brings new insights into the microscopic features of gingival orofacial granulomatosis, thus providing key elements to oral practitioners to ensure accurate and timely OFG diagnosis. The accurate diagnosis of OFG allows targeted management of symptoms and patient monitoring over time, along with early detection and treatment of extra-oral manifestations, such as Crohn’s disease.

## 1. Introduction

Orofacial granulomatosis (OFG) is a chronic inflammatory disease that is clinically characterized by lymphedema of the mouth and/or the face [1,2,3,4]. Lip edema is the most common finding (over 90%), although it is rarely the sole clinical feature. Oral examination may reveal cobblestoning, ulceration, tags, a fissured tongue, and chronic erythematous gingivitis [5,6], with frequently alternating acute inflammation and remission periods. A global prevalence of 0.8% has been suggested [7]. OFG is more frequently diagnosed in children and young adults (median age of diagnosis: 28 years [8,9]), although it may appear at any age and has no specific association with ethnicity or gender [10]. While most OFG features appear as isolated clinical entities, associations with extra-oral conditions, such as intestinal Crohn’s disease (CD), sarcoidosis, and Melkersson–Rosenthal syndrome [11], have also been reported. Microscopic evaluations of affected oral tissues emphasize the presence of chronic inflammation [12], with noncaseating granulomas as well as interstitial inflammatory lymphocytic infiltrate surrounding epithelioid histiocytes [3,13,14]. The etiology of OFG is currently poorly understood. Causes are expected to be largely multifactorial [3,13,15,16,17] with the potential participation of delayed hypersensitivity to food substances and food preservatives [5,18,19], microbial infections [20,21], inflammatory/immunologic dysregulation, genetics, and hereditary factors [1,7,22]. In addition, dental infections [23] and metals used for dental restorative materials (e.g., mercury [24,25,26], gold [25,27], cobalt and indium [28] constitutive of amalgam fillings and dental crowns) might also contribute to the initiation or worsening of OFG. The characterization of OFG in prepubertal patients remains to be depicted, especially after orthodontic therapies employing metal alloy devices, which are known to induce reactive or allergic inflammatory gingival pathologies that may lead to misdiagnosis. Moreover, first-line therapy is currently a matter of debate, with no single therapeutic alternative showing consistent efficacy or predictability [5]. While several treatments have been proposed, either alone or in combination [29,30,31,32,33,34], there is no clear consensus on the matter, and steroid-free treatments are rarely successful [35].

The aim of the present case report is to increase the awareness of oral practitioners about the importance of OFG diagnosis and to bring original insights into this condition using 2D/3D microscopy. In this context, the present paper describes the clinical and microscopic features of the gingival orofacial granulomatosis in an 8-year-old male patient after orthodontic therapy.

## 2. Case Report

### 2.1. Clinical Features

**Figure 1:**
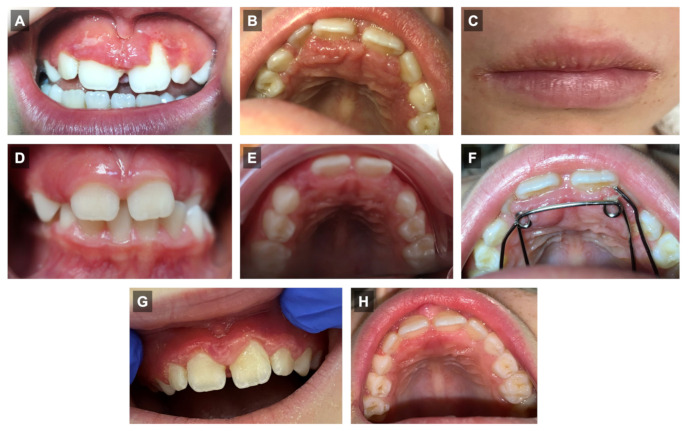
OFG clinical observations. (**A**–**C**) Gingival and labial lesions occurring after the placement of an interceptive orthodontic (quad-helix) appliance. Gingival examination before (**D**,**E**) and on the day of quad-helix placement (**F**). (**G**,**H**) Gingival examination 3 months after daily corticoid mouthwashes.

An 8-year-old child attended the dental department of Toulouse University Hospital for an evaluation of gingival lesions that occurred after the placement of an interceptive orthodontic (quad-helix) device. An intra-oral examination showed an erythematous hyperplasia of the palatal and buccal gingiva in the upper and lower jaws with a granular appearance exhibiting ulcerations (Figure 1A,B) in a moderate oral hygiene context. The peri-oral examination revealed a firm upper labial swelling and angular cheilitis. The patient’s mother reported that the labial swelling occurred intermittently (Figure 1C). Oral examinations before (Figure 1D,E) and on the day of the quad-helix placement (Figure 1F) did not reveal any gingival lesions; oral clinical signs appeared a few weeks after the placement of the orthodontic appliance. The patient did not report any known extra-oral diseases. The patient’s medical history did not reveal any known extra-oral diseases or allergies. No associated family medical history was reported. Facing such a clinical presentation, the hypothesis of orofacial granulomatosis was formulated. Inflamed gingival tissue between upper right central and lateral maxillary incisors was sampled using a #3 punch after local infiltration with 2% lidocaine/1:200,000 epinephrine. Microscopy investigations confirmed the diagnosis of gingival orofacial granulomatosis (see below, Histopathology and 3D microscopy analysis). A gastrointestinal examination and allergy (including quad-helix alloy) and blood tests did not highlight any disturbances with the exception of a weakly positive anti-*Saccharomyces cerevicae* IgG auto-antibody (ASCA), showing a value just above the threshold. After the removal of the orthodontic device, treatment included daily corticoid mouthwashes (prednisolone 20 mg tablet, diluted in a glass of water, 3 times per day) for 3 months, which resulted in a slight improvement in clinical signs (Figure 1G,H). Nevertheless, oral pain and gingival inflammation recurred once the treatment stopped.

### 2.2. Histopathology and 3D Microscopy Analysis

**Figure 2:**
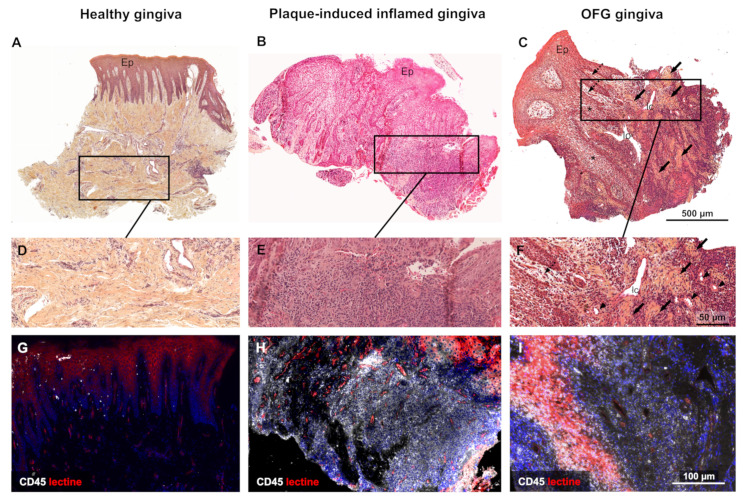

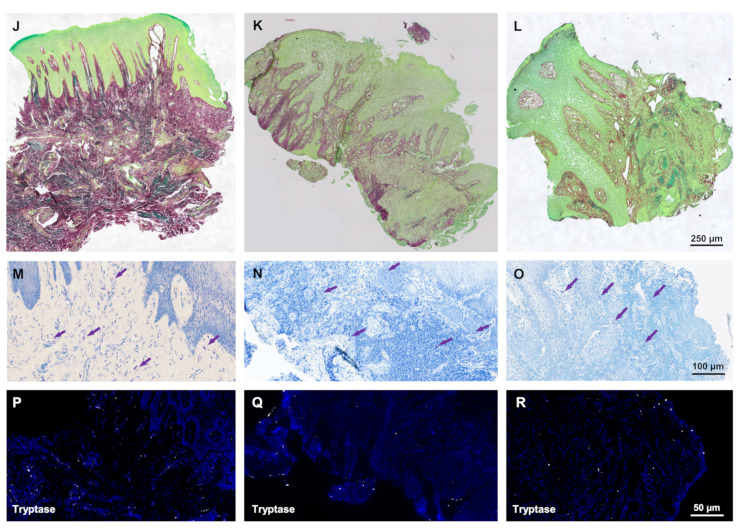
OFG histopathology. Healthy and plaque-induced inflamed gingiva were compared to gingival samples recovered from OFG tissue. The microscopic examination of the OFG tissue showed a papillomatous-like Malpighian parakeratinized epithelium with spongiosis (*; **C**). The underlying connective tissue showed numerous noncaseating microgranulomas with epithelioid histiocytes displaying reniform nuclei and multinuclear cells (black arrows; **C**,**F**). In the OFG tissue, intense, deep perivascular and interstitial but also intraepithelial lymphomacrophagic infiltrates were depicted. In comparison, plaque-induced inflamed gingiva lymphomacrophagic infiltrate was confined to deep connective tissue (**B**,**E**). Uncongestioned blood vessels (arrowheads; **F**) and lymphatic channels (lc; **C**,**F**) were highlighted in OFG connective tissue, displaying loose, edematous subepithelial stroma (dashed arrows; **C**,**F**). (**G**–**I**): Tissue localization of CD45 cells (white). The OFG gingival sample exhibited a strong epithelial lymphomacrophagic infiltrate (**I**) compared to plaque-induced inflamed gingiva, showing leucocyte infiltration only in the deep connective tissue, (**H**) while the healthy gingival sample displayed few leucocytes in the basal layer of the epithelial and subepithelial stroma distribution (**G**). The OFG connective tissue exhibited a more disorganized ECM with almost complete collagen loss (**L**) compared to plaque-induced inflamed gingiva (**K**). High-magnification TB (magenta arrows; **M**–**O**) and antitryptase immunofluorescence (white fluorescent cells; **P**–**R**) mast cells localization highlighted similar distributions in healthy (**M**,**P**), plaque-induced inflamed, (**N**,**Q**) and OFG gingival (**O**,**R**) samples. (**A**–**F**) hematoxylin-eosin staining. (**G**–**I**) CD45 immunofluorescence. (**J**–**L**) Red picrosirius (PS) staining. (**M**–**O**) Toluidine blue (TB) staining. (**P**–**R**) anti-tryptase immunofluorescence. Cell nuclei stained by DAPI; epithelium (Ep) and blood vessels localized using human biotinylated lectin revealed by the Alexa 647–streptavidin conjugate (**G**–**I**,**P**–**R**).

Microscopy investigations of the affected gingiva were performed by histochemistry to investigate the epithelial and connective cells (including mast cells) and the extracellular matrix (ECM). CD45 (ab10558 Abcam^®^, 1:100) and tryptase (ab2378 Abcam^®^, 1:100) immunostaining were carried out for pan-leucocyte and mast cell detection, respectively. Plaque-induced inflamed and healthy tissues, matched to the OFG sample for age and sex, were used as controls. The OFG gingival sample microscopic investigations showed typical OFG features (Figure 2). In contrast to healthy (Figure 2A,D,G) and plaque-induced inflamed (Figure 2B,E,H) gingiva, OFG gingival tissue exhibited a papillomatous-like epithelium with spongiosis and noncaseating microgranulomas containing epithelioid histiocytes and multinuclear cells within the connective tissue (Figure 2C,F,I). OFG connective tissue also exhibited intense interstitial and intraepithelial lympho-macrophagic infiltrate, together with numerous uncongested blood vessels and dilated lymphatic vessels. OFG conjunctival papilla displayed loose, edematous subepithelial stroma (Figure 2C,F). In contrast to healthy and plaque-induced inflamed gingival controls, the OFG tissue had a strongly disorganized ECM with almost complete collagen loss (Figure 2J,K,L, Supplementary Figure S1). All samples showed a similar mast cell distribution (Figure 2M–R, Supplementary Figure S2).

**Figure 3:**
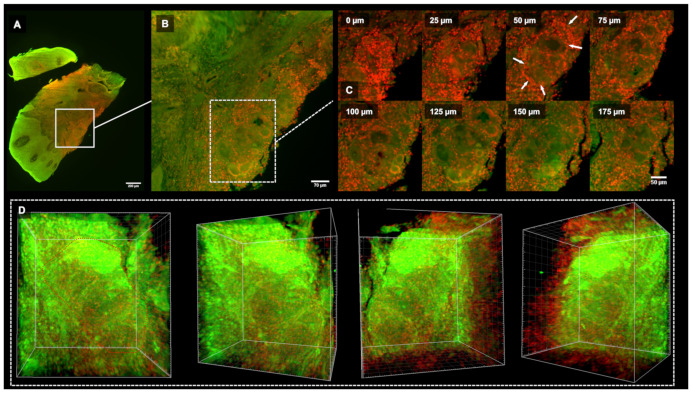
Gingival OFG 3D microscopy. (**A**): Low magnification LSFM acquisition. (**B**): Granuloma focus. (**C**): Granuloma 175 µm depth series acquisition. Note the leucocytes cells surrounding the granuloma edges (white arrows). (**D**): Sequential 3D frames emphasized the granuloma multilocular oval-shape (Supplementary Movie S1).

For the 3D microscopy, samples were fixed using 3.7% PFA, and optical clearing was performed with Benzyl Alcohol 2vol Benzyl Benzoate solution. Then, the tissues were stained overnight by propidium iodide (Sigma-Aldrich; 10 µg/mL with RNase). The 3D acquisitions were carried out using LightSheet Z7–Zeiss^®^ and analyzed using Imaris^®^ software. They confirmed the 2D OFG gingival histopathology, including strong leucocyte connective tissue infiltration together with oval-shaped, multilocular granulomas (Figure 3, Supplementary Figure S3, Supplementary Movies S1–S4).

## 3. Discussion

OFG assessment in gingival location is often a diagnosis of exclusion since various medical contexts can produce similar clinical features, such as foreign body reactions, mycobacterial infections (like chronic candidiasis), erosive lichen, desquamative gingivitis, bullous lesions or gingival inflammation following a worsening of oral hygiene [2,14,36,37]. Here, the hypothesis of plaque-induced gingivitis has been discarded after execution of professional oral cleaning and following careful monitoring of patient’s oral hygiene (to exclude gingival inflammation driven by the installation of orthodontic device [13]). As such, a diagnosis of OFG has been considered given the presence of its typical clinical evidences, including lip edema [4]. To confirm the OFG diagnosis, histopathological investigations have been carried out. A direct comparison with plaque-induced inflamed and healthy gingiva specimens highlighted OFG-specific gingival features including noncaseous granulomas surrounded by a lymphoplasmacytic and macrophagic infiltrate. Perivascular lymphocytic infiltrate, usually observed in OFG [17], has also been detected in deep connective tissue. In this subject, intense leucocytic infiltrate was also identified in epithelial layers. In addition, epithelium spongiosis and underlying connective tissue edema (usually absent in plaque-induced gingivitis) have also been detected, although OFG epithelial changes are unspecific [13]. Histopathological features of OFG granulomas are very similar to those encountered in intestinal Crohn’s disease (CD) [3]. As a matter of fact, a large number of OFG patients (approximately 40% of children and 20–50% of adults) are diagnosed with CD [2,38]. In the reported case, ASCA level suggested that OFG could be an inaugural sign of CD. Since OFG can precede by several years the onset of intestinal symptoms [39], long-term monitoring of pediatric patients is of paramount importance to provide a timely diagnosis of gastrointestinal involvement. A long-term follow-up of our patient will be carried out together with additional medical investigations to monitor the development of any extra-oral manifestation. To date, the OFG etiology remains unclear [40]. Patients with OFG have a higher atopy incidence compared with general population [17]. Hay fever, atopic eczema, asthma or oral allergy syndrome have been reported as affecting nearly 80% of OFG patients compared to 15% in the general population [11]. The literature reports cases of OFG associated with hypersensitivity to food substances and food preservatives (like cinnamon or benzoates compounds [5,18,19]), and to dental restorative materials [24,25,26,27,41], but only one author has described the relationship between OFG and orthodontic appliance [42]. Our paper deals with a gingival OFG occurrence following the application of an interceptive orthodontic device in a prepubertal, healthy patient, without known allergies or oral signs prior to the initiation of the orthodontic therapy. Allergy skin and oral mucosa tests did not highlight hypersensitivity to orthodontic appliance alloy. The orthodontic device in an irritant local context might have initiated the OFG emergence, persisting even after removal of the orthodontic appliance. Treatment of local OFG inflammation and pain is often challenging and non-rewarding. It is, therefore, important to provide clear information to the patient regarding the chronic nature of OFG and its possible refractory behavior. OFG can pursue an acute, recurrent, or chronic course, with low probability of a complete remission [14]. Here, although symptoms improved after removal of the appliance and local administration of corticosteroids, no complete resolution of the lesions has been obtained.

The originality of this case-report lies in the OFG clinical description on a pre-pubertal child following the application of an interceptive orthodontic device, the unique microscopy investigation through combined 2D/3D histopathology. Furthermore, comparison with plaque-induced inflamed and healthy gingiva specimens supports differential diagnosis based on histological differences between OGF, gingivitis and healthy conditions. Moreover, the rich clinical and microscopic iconographic documentation would provide important clues to guide the clinician’s investigations.

## 4. Conclusions

This article sheds light on a rare and yet ill-known condition, the OFG, which can however be found in the daily practice of pediatric dentists or orthodontists. Oral practitioners should be aware of the possible implication of orthodontic appliances in the onset of such a condition. They also have a key role in recognizing the major OFG clinical manifestations or at least in raising OFG as a differential diagnosis, especially regarding plaque-induced gingivitis, in order to allows early symptoms’ management. Given the potential relationship between OFG in childhood and extraoral diseases, children with OFG should always be referred to pediatrician, allergologist and dermatologist for further examination and follow-up.

## Data Availability

Not applicable.

## Supplementary Materials

The following supporting information can be downloaded at: https://zenodo.org/record/7609344, Figure S1: Polarized acquisition of red picrosirius staining; Figure S2: Mast cells localization by anti-tryptase immunofluorescence; Figure S3: Healthy gingiva 3D microscopy; Movie S1: 3D OFG gingival granulomas movie (12×); Movie S2: Whole LSFM stack acquisition movie of gingival OFG sample (5×); Movie S3: LSFM OFG gingival focus stack acquisition movie (12×); Movie S4: LSFM OFG gingival granuloma focus stack acquisition movie.

## Abbreviations

The following abbreviations are used in this manuscript:

ASCA	*anti-Saccharomyces cerevicae* IgG auto-antibody
CD	Crohn’s disease
ECM	Extra-Cellular Matrix
OFG	Orofacial granulomatosis
